# Interactions and substrate selectivity within the SctRST complex of the type III secretion system of enteropathogenic *Escherichia coli*

**DOI:** 10.1080/19490976.2021.2013763

**Published:** 2021-12-29

**Authors:** Irit Tseytin, Shir Lezerovich, Nofar David, Neta Sal-Man

**Affiliations:** The Shraga Segal Department of Microbiology, Immunology and Genetics, Faculty of Health Sciences, Ben-Gurion University of the Negev, Beer-Sheva, Israel

**Keywords:** Type 3 section system, export apparatus, SctRST complex, transmembrane domain, oligomerization

## Abstract

Many bacterial pathogens employ a protein complex, termed the type III secretion system (T3SS), to inject bacterial effectors into host cells. These effectors manipulate various cellular processes to promote bacterial growth and survival. The T3SS complex adopts a nano-syringe shape that is assembled across the bacterial membranes, with an extracellular needle extending toward the host cell membrane. The assembly of the T3SS is initiated by the association of three proteins, known as SctR, SctS, and SctT, which create an entry portal to the translocation channel within the bacterial inner membrane. Using the T3SS of enteropathogenic *Escherichia coli*, we investigated, by mutational and functional analyses, the role of two structural construction sites formed within the SctRST complex and revealed that they are mutation-resistant components that are likely to act as seals preventing leakage of ions and metabolites rather than as substrate gates. In addition, we identified two residues in the SctS protein, Pro23, and Lys54, that are critical for the proper activity of the T3SS. We propose that Pro23 is critical for the physical orientation of the SctS transmembrane domains that create the tip of the SctRST complex and for their positioning with regard to other T3SS substructures. Surprisingly, we found that SctS Lys54, which was previously suggested to mediate the SctS self-oligomerization, is critical for T3SS activity due to its essential role in SctS-SctT hetero-interactions.

## Introduction

Type III secretion systems (T3SSs) are syringe-shaped nanocomplexes responsible for the transfer of effector proteins (virulence factors) into host cells. These complexes are found in a wide variety of Gram-negative bacterial pathogens, including *Escherichia coli*, and species of *Yersinia, Shigella, Salmonella*, and *Pseudomonas*.^1^The translocated effectors manipulate key cellular pathways that ultimately promote colonization of the host and bacterial survival.^2,3^ In evolutionary terms, the T3SS is related to the bacterial flagellum, and many structural components are highly conserved between these two systems. The T3SS complex is comprised of more than 20 different proteins, most of which are found in multiple copies. The complex spans the inner and outer bacterial membranes and has a protruding extracellular needle extending outwards to the host cell membrane.

**Figure 1.**
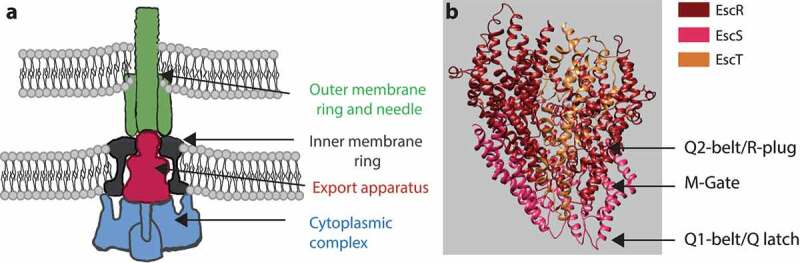
**Side view of the T3SS. (A)** The T3SS components assemble across the inner and outer membranes. The cytoplasmic complex is shown in blue, the export apparatus in dark red, the inner-membrane ring in grey, and the outer-membrane ring and needle in green. **(B)** The core structure of the export apparatus forms three constriction sites: the Q1-belt/Q latch, the M-gate, and the Q2-belt/R-plug

Among the most highly conserved substructures within the T3SS complex is the export apparatus (Figure 1A), which is found at the center of the inner membrane ring, facing the cytoplasmic complex, and which acts as the entry portal for T3SS-transported substrates.^3,4^ The export apparatus is composed of five membrane-associated proteins, SctR, SctS, SctT, SctU, and SctV, named according to the unified Sct (secretion and cellular translocation) system.^3,5^ These proteins assemble in a stoichiometry of 5:4:1:1:9 to form a funnel-shaped structure that connects the inner rod and the needle proteins on its wider end to the inner membrane ring on its narrower end.^6–9^ The proteins of the export apparatus are essential for type III secretion (T3S), and it is commonly held that they initiate the assembly process of the T3SS complex.^10–14^

The internal core of the T3SS export apparatus, which forms the translocation channel, is composed of three proteins, SctR, SctS, and SctT, which form a pseudohexameric helical structure with multiple narrowing points.^7–9^ These include a Q1-belt/Q-latch, formed by conserved Gln residues along a loop in SctS, an M-gate, formed by the highly conserved Met-Met-Met loop in the SctR sequence, and a Q2-belt/R-plug, formed by the large hydrophobic loop of SctT (Figure 1B). It was recently shown that the M-gate forms the main constriction site of the export apparatus, and that it opens only slightly upon substrate entry into the channel, thereby probably preventing leakage of ions and small molecules through the channel and preserving the membrane barrier.^9^ This observation is in keeping with findings that mutations within the conserved methionine loop of SctR result in reduced bacterial fitness and viability.^9,15^ The Q1 – and Q2-belts flank the M-gate, with the Q1-belt enabling side-chain independent substrate transport and the Q2-belt acting as a flexible lid that opens up to allow the entry of substrates into the atrium of the T3SS channel. While it has been reported that some mutations in the Q1- and Q2-belts disturb T3SS function,^9,16^ an examination of the available structural data gives rise to the question whether the Q-belts play an active or a passive role in substrate gating.

To address the above question and to identify additional residues within the SctRST complex that are critical for T3SS function, we set out to subject the complex to extensive mutagenesis. To this end, we used the T3SS of enteropathogenic *E. coli* (EPEC), one of the causative agents of pediatric diarrhea,^17^ as our model system. We therefore use the EscRST nomenclature throughout the manuscript. Overall, our results suggest that the Q1- and Q2-belts are non-selective components in the transport process and that they probably act as seals – preventing leakage of ions and metabolites – rather than acting as substrate gates. We also show that Pro23 and Lys54 of the EscS protein are critical for the proper activity of the complex, due to their involvement in protein–protein interactions within the EscRST complex and in the physical orientation of the transmembrane domains (TMDs) of EscS within the EscRST complex.

## Results

### The conserved glutamines in the Q1-belt are not essential for T3SS activity

**Figure 2.**
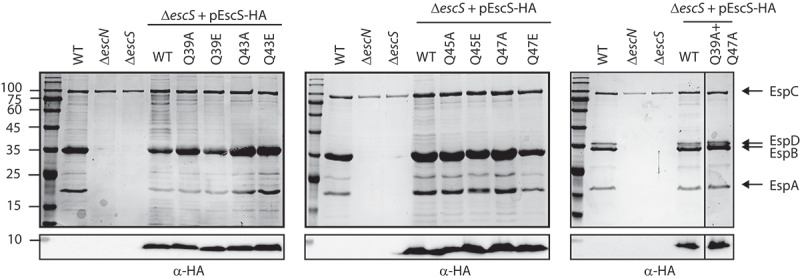
**Mutations in the Q1-belt have no effect on T3SS activity**. Protein secretion profiles of wild-type (WT) EPEC, Δ*escN*, Δ*escS*, and Δ*escS* transformed with plasmids bearing the WT sequence of *escS* or containing single and double mutations as indicated. The strains were grown under T3SS-inducing conditions, and protein expression was induced with IPTG. The secreted fractions were normalized and filtered, and the protein contents of the concentrated the supernatants were analyzed by 12% SDS-PAGE with Coomassie staining (upper panels). The T3SS-secreted translocators, EspA, EspB, and EspD, are marked on the right of the gel. Also indicated is the location of EspC, which is not secreted via the T3SS. For the Δ*escN* and Δ*escS* strains, no T3SS activity was observed. In the Δ*escS* strain transformed with pEscS_wt_-HA, T3S was restored, and none of the glutamine point mutations led to reduced secretion. The plasmid-expressed EscS-HA variants were identified by analyzing the bacterial pellets on SDS-PAGE, followed by western blot analysis with an anti-HA antibody (lower panels)

Sequence alignment of EscS with its homologs, FliQ of the *Salmonella* flagellum, YscS of the *Yersinia* T3SS, Spa9 of the *Shigella* T3SS, and SpaQ of the *Salmonella* SPI-1 T3SS, showed that four glutamines (at positions Gln39, Gln43, Gln45 and Gln47) are highly conserved.^18^ These glutamines form a Q1-belt at the tip of the export apparatus that serves as the substrate entry site.^9^ To examine whether the conserved glutamines within the Q1-belt of EPEC are essential for T3SS activity, we mutated single glutamine residues of EscS to either non-polar (alanine) or highly polar (glutamic acid) residues. Vector-expressed EscS-HA variants that encode either the EscS WT sequence or EscS with point mutations were transferred into EPEC Δ*escS*, and their ability to complement T3SS activity was examined. T3SS activity was assessed in terms of the ability of the EPEC strains, grown under T3SS-inducing conditions, to secrete three T3SS translocators (EspA, EspB, and EspD) into the culture supernatant. WT EPEC demonstrated T3SS activity, while the Δ*escS* mutant strain did not secrete translocators and displayed a secretion pattern similar to that of the EPEC Δ*escN* strain, which is deleted for the T3SS ATPase gene (Figure 2). Transformation of EPEC Δ*escS* with plasmids that encode EscS-HA with various glutamine point mutations completely restored T3SS activity, regardless of the nature of the mutation (position or side chain). A similar result was obtained for a double-mutant EscS protein, in which two glutamines, Gln39 and Gln47, had been replaced with alanines (Figure 2). While this mutational analysis is not complete and is based only on single substitutions and on one double mutation, our results suggest that there is redundancy in the glutamines of the Q1-belt and that while their presence is collectively important (probably to provide an aqueous environment and to support cargo engagement), they are not critical for T3SS activity, as shown by the lack of effect of the mutations at one or two positions.

### Point mutations in the EscT plug (Q2-belt) preserve T3SS activity

**Figure 3.**
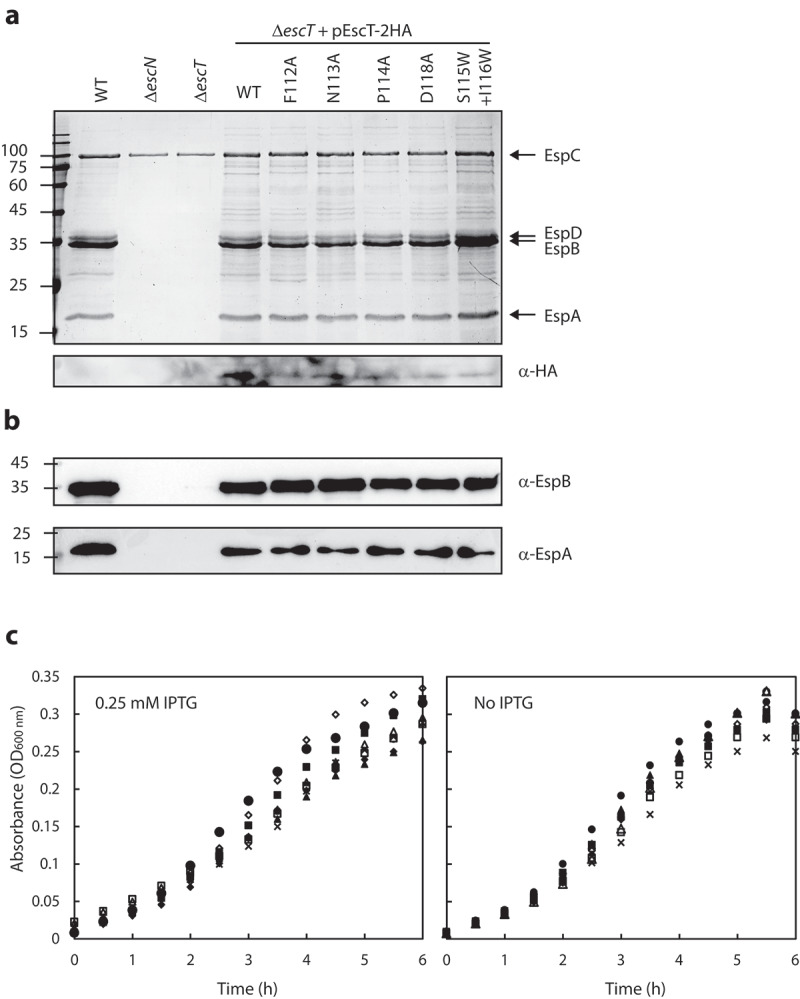
**Mutations in the EscT Q2-belt have no effect on T3SS substrate gating. (A)** Protein secretion profiles of WT EPEC, Δ*escN*, Δ*escT*, and Δ*escT* transformed with pEscT_wt_-2HA, EscT_F112A_-2HA, EscT_N113A_-2HA, EscT_P114A_-2HA, EscT_D118A_-2HA and pEscT_S115W+I116W_-2HA (upper panel). The secreted fractions were obtained using a protocol similar to that described in the legend to Figure 2. The expression of EscT-2HA variants was assessed by analyzing the bacterial pellets on SDS-PAGE, followed by western blot analysis with an anti-HA antibody (lower panel). **(B)** The secretion levels of the EspA and EspB translocators were determined by analyzing the bacterial supernatant by western blot analysis with anti-EspA and anti-EspB antibodies. **(C)** Growth curves of WT EPEC (○), Δ*escN* (■), Δ*escT* (●), and Δ*escT* transformed with plasmid-expressed EscT-2 HA WT (□), F112A mutation (▲), N113A mutation (Δ), P114A mutation (◆), D118A mutation (◇), and the double S115W and I116W mutation (˟). Bacteria were grown at 37°C in DMEM with 0.25 mM IPTG (left panel) or no IPTG (right panel). Optical density at 600 nm was determined every 30min

It was previously shown that the FliR protein of the *Salmonella* flagellum contains a structural loop that is composed of large hydrophobic residues and that completely occludes the T3SS channel, and it was therefore termed a R plug/Q2-belt.^8^ Recently, a homologous structural element of the SpaR protein of *Salmonella* T3SS was shown to undergo a conformational switch to allow effector protein secretion through the T3SS channel.^9^ To examine whether EscT forms a similar dynamic element in the EscRST complex, we examined the effect of various point mutations in the EscT Q2-belt. Based on a 3D model of the EscRST structure,^18^ we chose to mutate residues Phe112, Asn113, Pro114 and Asp118, which are situated at the center of the belt and may therefore play a critical role in the belt’s conformation and function. Vectors encoding WT and mutant variants of EscT-2 HA were transformed into ∆*escT*, and their ability to complement ∆*escT* T3SS activity was examined. We found that expression of EscT_wt_-2HA within Δ*escT* restored T3SS activity, as did the expression of EscT_F112A_-2HA, EscT_N113A_-2HA, EscT_P114A_-2HA, and EscT_D118A_-2HA (Figure 3A). Expression of the labeled EscT proteins was confirmed by analyzing bacterial samples by SDS-PAGE and western blot with anti-HA antibody (Figure 3A). These results suggested that none of the investigated residues, as an individual residue, is crucial for the function of EscT. However, the above experiment did not enable us to assess the effect of multiple changes in the Q2-belt region. To examine whether a more extensive change in the EscT Q2-belt would disrupt proper T3SS functioning, we generated a double mutant in which two residues, Ser115 and Ile116, were replaced with bulky tryptophan residues (EscT_S115W+I116W_-2 HA). The plasmid was transformed into the Δ*escT* strain, and T3SS activity was assessed. It was found that expression of EscT_S115W+I116W_-2HA fully restored the T3SS of Δ*escT* (Figure 3A).

To evaluate whether the hierarchy of secretion had been altered due to the mutations in the EscT Q2-belt, we examined the secretion levels of two translocators, EspB and EspA. In correlation with the Coomassie staining, we observed similar EspB and EspA levels in EscT WT and mutant forms (Figure 3B), which indicated that the mutations did not have an effect on the transition from secretion of translocators to secretion of effectors. To examine whether expression of mutated EscT affected bacterial fitness, due to a ‘leaky’ T3SS complex, we cultured WT EPEC, Δ*escN*, Δ*escT*, and Δ*escT* strains expressing WT or mutant forms of EscT-2HA under T3SS-inducing conditions. To evaluate the effect of EscT on bacterial fitness, the strains were grown in the absence or presence of 0.25 mM IPTG. We observed only a minimal effect of the mutations on the growth rates of the bacteria (Figure 3C). These results thus suggest that mutations in the EscT Q2-belt do not contribute to higher membrane permeability and therefore do not impose a fitness cost.

### Pro23 and Lys54 point mutations abolish EPEC T3SS activity

**Figure 4.**
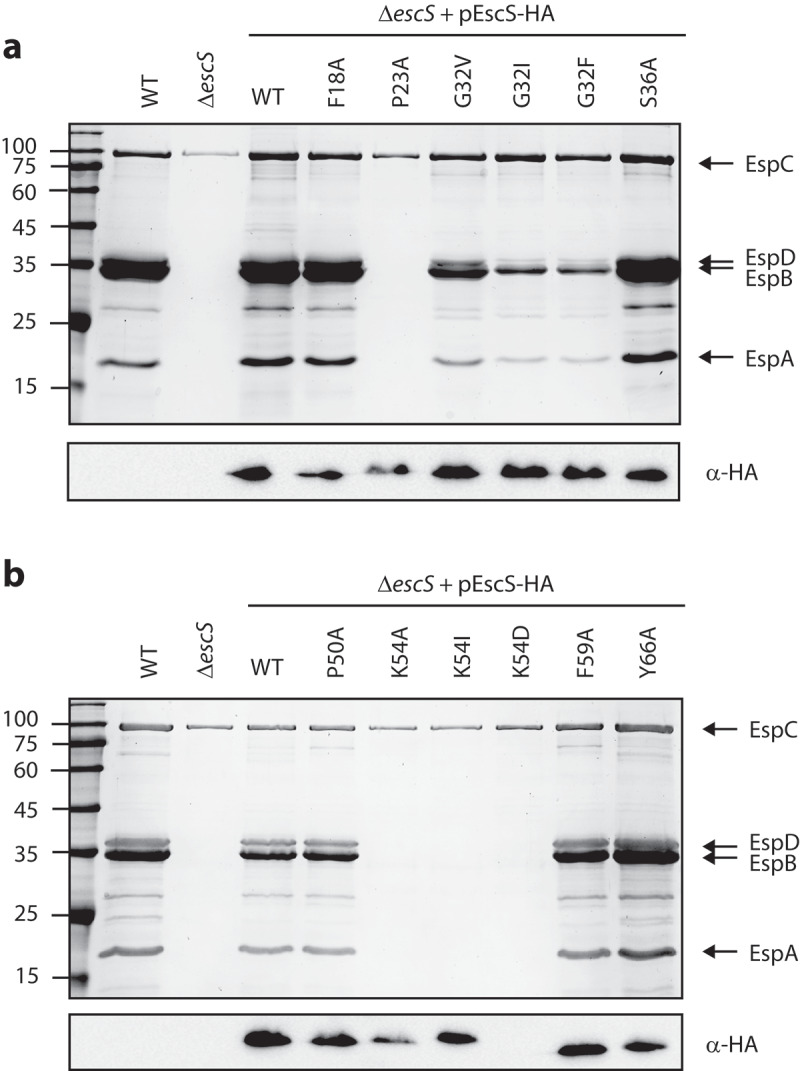
**Effect of point mutations in EscS TMDs on EPEC T3SS activity. (A)** The effect of point mutations in EscS TMD1 was assessed by analyzing the protein secretion profiles of WT EPEC, Δ*escS*, and Δ*escS* transformed with pEscS_wt_-HA, EscS_F18A_-HA, EscS_P23A_-HA, EscS_G32V_-HA, EscS_G32I_-HA, EscS_G32F_-HA, and EscS_S36A_-HA (upper panel). The secreted fractions were obtained using a protocol similar to that described in the legend to Figure 2. The expression of EscS-HA variants was determined by analyzing the bacterial pellets on SDS-PAGE, followed by western blot analysis with an anti-HA antibody (lower panel). **(B)** The effect of point mutations in EscS TMD2 was assessed similarly to that of point mutations in EscS TMD1, as shown in panel A

In our previous work, we found that replacement of both EscS TMDs by an alternative hydrophobic sequence failed to complement T3SS in the Δ*escS* strain and generated a similar secretion pattern to that of the Δ*escN* mutant strain.^18^ In addition, we showed that a Lys to Ala mutation at position 54 impaired T3SS activity.^18^ To pinpoint the specific amino acids in the EscS TMDs that are critical for the protein function within the T3SS complex, we mutated several amino acid residues in EscS TMD1 and TMD2 that constitute part of known TMD motifs or that are aromatic, polar, or helix-breaker residues. These included mutations of Gly32 and Ser36 [which belong to a GxxxG-like motif, the best-characterized motif for TMD–TMD interactions ^19,20^], of aromatic residues (Phe18, Phe59, and Tyr66), of a polar residue (Lys54), and of the helix-breakers (Pro23 and Pro50), all of which have been reported to be involved in protein-protein interactions within the membranes.^21–24^ The amino acids Ser36, Phe18, Phe59, Tyr66, Lys54, Pro23, and Pro50 were mutated to the small non-polar alanine, while Gly32 was mutated to a large non-polar residue (valine, isoleucine, or phenylalanine) to induce a large structural change. Vectors expressing various EscS-HA mutations were transferred into Δ*escS* to examine their ability to complement T3SS activity. It was found that mutations P23A in TMD1 and K54A in TMD2 completely abolished T3SS activity, while G32I and G32F in TMD1 decreased the T3S level and mutations F18A, G32V, S36A, P50A, F59A, and Y66A had no effect on T3SS activity (Figure 4). To examine whether the disruption of T3SS activity by the K54A mutation was due to the loss of a positively charged residue or to the change from a large to a small residue, we mutated Lys54 to isoleucine (large and non-polar) or aspartic acid (large and polar). An examination of the T3SS activity of these mutations demonstrated similar disruption of the T3SS activity (Figure 4B), thus suggesting that Lys54 is critical for EscS function by virtue of its charge. To confirm that the defective function of the EscS mutants was not due to a protein expression issue, we subjected whole-cell lysates (WCLs) to SDS-PAGE and western blot analysis with an anti-HA antibody. We observed similar expression levels for all EscS mutants, except for EscS_K54D_-HA, which showed no protein expression (Figure 4). Overall, our results suggest that Pro23 and Lys54 are critical for the proper activity of EscS, probably due to their involvement in protein–protein interactions or in the physical orientation of EscS within the EscRST complex.

### EscS Lys54 is not essential for self-interactions

**Figure 5.**
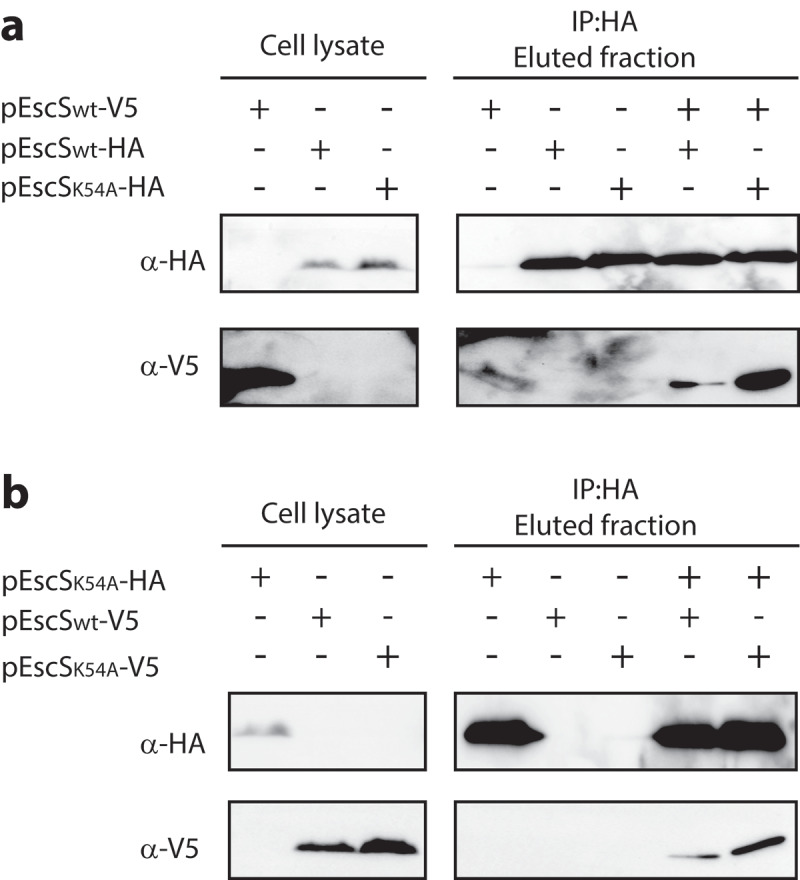
**EscS Lys54 is not involved in EscS self-association*in vitro***. Whole-cell lysates of *E. coli* BL21 (λDE3) expressing EscS_wt_-V5, EscS_wt_-HA, EscS_K54A_-HA or EscS_K54A_-V5 were subjected to immunoprecipitation using protein-G beads linked to an anti-HA antibody. The bacterial lysates were incubated each on its own or each in combination with an additional lysate. Whole-cell lysates and elution fractions were separated on a 12% SDS-PAGE and analyzed by western blotting with anti-HA and anti-V5 antibodies. **(A)** EscS_wt_-V5 co-eluted with both EscS_wt_-HA and EscS_K54A_-HA. **(B)** EscS_wt_-V5 and EscS_K54A_-V5 co-eluted with EscS_K54A_-HA

The conserved Lys54 was previously shown to form a salt bridge between neighboring subunits of FliQ.^8^ As we previously showed that EscS can self-interact, thereby forming a homodimer or possibly a higher order homo-oligomer,^18^ it seemed likely that Lys54 of EscS could be involved in this protein self-interaction. Using a co-immunoprecipitation assay, we examined the effect of the K54A mutation on the ability of EscS to self-interact *in vitro*. We found that EscS_wt_-V5 co-eluted with both EscS_K54A_-HA and EscS_wt_-HA (Figure 5A), and, moreover, that EscS_K54A_-HA co-eluted with similar levels of EscS_wt_-V5 and EscS_K54A_-V5 (Figure 5B). These results suggest that Lys54 of EscS is not essential for the interaction between EscS subunits *in vitro*.

### EscS Lys54 is critical for the EscS-EscT interaction within the EscRST complex

**Figure 6.**
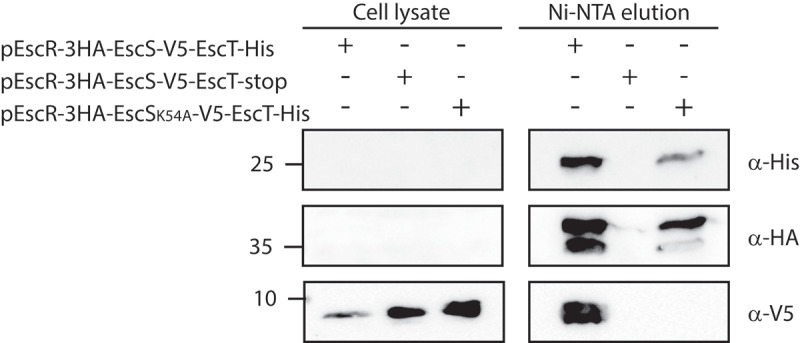
**EscS Lys54 is critical for the EscS-EscT interaction within the EscRST complex**. Whole-cell lysates of *E. coli* BL21 (λDE3) expressing EscR-3HA/EscS-V5/EscT-His, EscR-3HA/EscS_K54A_-V5/EscT-His or EscR-3HA/EscS-V5/EscT were incubated overnight with Ni-NTA beads. The beads were then washed, and interacting proteins were eluted and loaded onto 12% SDS-PAGE. The samples were analyzed by western blotting with anti-His, anti-HA and anti-V5 antibodies

We then examined whether the K54A mutation in EscS interferes with the formation of a proper export apparatus complex. For that purpose, we cloned the genes encoding the core export apparatus proteins, *escR/escS/escT*, on a single plasmid using their native genetic orientation, while labeling them with different tags for protein detection. Such a co-expression design was previously reported to yield higher expression levels of EscR, EscS, and EscT homologs and to stabilize the export apparatus complex.^8,25,26^ Using the above-described plasmid, we examined the interaction between EscR, EscS, and EscT proteins expressed in *E. coli* BL21. To determine whether the K54A mutation disrupts interactions within the EscRST complex, we pulled down EscT-His, expressed from either pEscR-3HA/EscS-V5/EscT-His or pEscR-3HA/EscS_K54A_-V5/EscT-His vectors, and analyzed its interacting partners. As the negative control, we used a vector that expressed a non-labeled EscT protein (pEscR-3HA/EscS-V5/EscT). Samples of WCLs and elution fractions, corresponding to EscT interacting partners, were loaded on 12% SDS-PAGE and then analyzed by western blotting with anti-His, anti-HA and anti-V5 antibodies. We found that EscR-3HA and EscS_wt_-V5 co-eluted with EscT-His (Figure 6), thus confirming the formation of the core export apparatus complex *in vivo*. Pull-down elution of an *E. coli* strain expressing unlabeled EscT excluded the possibility that EscR-3HA and EscS_wt_-V5 proteins interact non-specifically with the Ni-NTA beads (Figure 6). These results suggest that the EscRST complex is formed independently of the other T3SS components. Moreover, when the EscRST complex contained a K54A mutation in the EscS protein, we observed co-elution of the EscR-3HA protein, but EscS_K54A_-HA was absent (Figure 6). Taken together, these results suggest that Lys54 is essential for EscS-EscT interaction within the EscRST complex.

To study whether Lys54 is involved in EscS-EscT interactions when expressed in the presence of the complete T3SS complex, we transformed the pEscRST plasmid into EPEC strains. To assess the functionality of the EscRST proteins, we first transformed the plasmid into EPEC ΔescR, ΔescS and ΔescT and then examined whether the strains restored T3SS activity. We observed T3SS activity in all the EPEC mutant strains that were transformed with the pEscRST vector, except for ΔescN, thus confirming that the labeled EscRST proteins were functional (Supplemental Figure 1).

**Figure 7.**
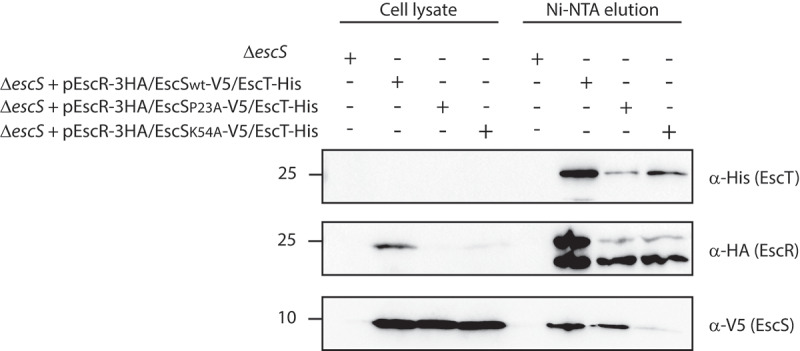
**EscS Lys54 is involved in hetero-interactions within the EscRST complex**. Whole-cell lysates of EPEC Δ*escS* strain transformed with the triple-protein expressing plasmids (pEscRST), pEscR-3HA-EscS-V5-EscT-His, pEscR-3HA-EscS_P23A_-V5-EscT-His or pEscR-3HA-EscS_K54A_-V5-EscT-His, were incubated overnight with Ni-NTA beads. The beads were then washed, and interacting proteins were eluted with 500 mM imidazole to preserve the native conditions. The eluted fractions were mixed with SDS-sample buffer and subjected to SDS-PAGE. Western blots were analyzed with anti-His, anti-HA and anti-V5 antibodies

The next step was to examine the interactions between EscR, EscS, and EscT proteins when expressed together with the other T3SS components. Analysis of the pulled-down fraction of the EscT-His protein confirmed that EscRST interactions were stable within the complete T3SS complex (Figure 7). To examine the interactions between the EscRST proteins when EscS carries the K54A mutation, we transformed pEscRS_K54A_T into the EPEC Δ*escS* strain. The pEscRS_P23A_T plasmid, which carries the P23A mutation in EscS and which showed a similar T3SS activity phenotype to K54A (Figure 4), was also examined. While both EscR-3HA and EscS_P23A_-V5 were detected in the elution fraction of pEscRS_P23A_T, EscR-3HA was detected in the elution fraction of pEscRS_K54A_T but only at a very low level of EscS_K54A_-V5 (Figure 7). These results provide further support for our previous findings that Lys54 is crucial for EscS-EscT interaction, even in the presence of the entire T3SS complex.

### Asp46 of EscS is involved in the regulation of substrate secretion

**Figure 8.**
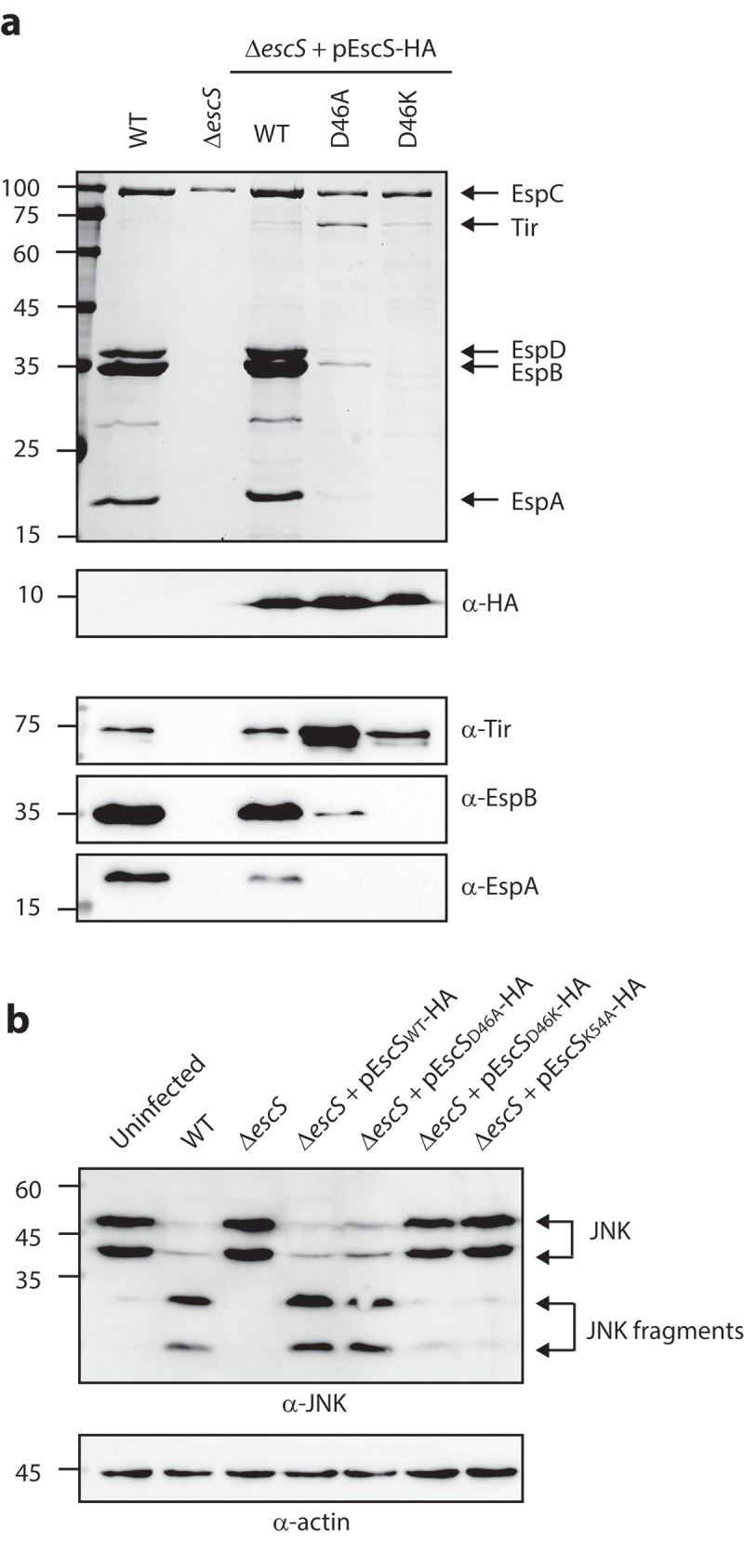
**Point mutations of Asp46 in EscS result in impaired T3SS activity and regulation. (A)** Protein secretion profiles of WT EPEC, Δ*escS*, and Δ*escS* expressing EscS_wt_-HA, EscS_D46A_-HA, or EscS_D46K_-HA. The secreted fractions were obtained using a protocol similar to that described in the legend to Figure 2. The expression of EscS-HA variants was determined by analyzing bacterial pellets on SDS-PAGE, followed by western blot analysis with an anti-HA antibody. The Δ*escS* strain expressing the WT EscS sequence (EscS_wt_-HA) restored T3S, while expression of EscS with a D46A point mutation resulted in dysregulated T3S, and expression of EscS with D46K abolished T3S. **(B)** Proteins extracted from HeLa cells infected with WT, Δ*escS*, or Δ*escS* expressing EscS_wt_-HA, EscS_D46A_-HA, EscS_D46K_-HA, or EscS_K54A_-HA were subjected to western blot analysis using anti-JNK and anti-actin (loading control) antibodies. JNK isoforms and their degradation fragments are indicated on the right of the gel

It has been suggested that the involvement of Lys54 in inter-subunit interactions of EscS homologs takes place via the formation of salt bridges between Lys54 and Glu/Asp46.^8,27^ Since our results had suggested that Lys54 is not essential for EscS self-interactions (Figure 5), we investigated the role of the conserved Asp46 in EscS function. To this end, we mutated Asp46 of the vector-expressed EscS-HA to either alanine or lysine and examined the ability of the two variants to restore T3SS activity. We found that the EPEC Δ*escS* strain expressing EscS_D46K_-HA showed no T3SS activity, while expression of EscS_D46A_-HA altered the regulation of T3SS substrate secretion, as reflected in reduced secretion of the translocators, EspA, EspB, and EspD, and hypersecretion of the translocated intimin receptor (Tir) (Figure 8A). Tir is the first effector to be secreted, and it should remain largely within the bacterial cells in the absence of host contact. To better detect the effect of the Asp46 mutation on substrate secretion, we analyzed the bacterial supernatants by western blot analysis with anti-Tir, anti-EspB, and anti-EspA antibodies. The analysis demonstrated a secretion defect of D46K and altered substrate secretion of D46A (Figure 8A). Similar phenotypes were observed for D46A and D46K mutations in the pEscRST vector (Supplemental Figure 2). We then created a mutant with complementary changes in the charged residues, namely, the double D46K and K54D mutant, to evaluate the contribution of the salt bridges to the assembly and function of the complex. However, we could not detect expression of this mutant variant (Supplementary Figure 3). To assess the functionality of EscS-HA mutant strains in a bacterial infection model, we examined the ability of EPEC Δ*escS* transformed with pEscS_wt_-HA, pEscS_D46A_-HA, pEscS_D46K_-HA, or pEscS_K54A_-HA to infect HeLa cells and to facilitate the translocation of effectors into the host cells. To this end, we infected HeLa cells with various EPEC strains (WT, Δ*escS*, and Δ*escS* complemented with pEscS-HA) and examined the cleavage pattern of JNK, a host protein that is cleaved by a translocated EPEC effector known as NleD.^28^ As expected, WT EPEC induced extensive degradation of JNK, relative to the uninfected sample and to the samples infected with Δ*escS* mutant strains (Figure 8B). EPEC Δ*escS* transformed with pEscS_wt_-HA or pEscS_D46A_-HA showed a JNK degradation profile similar to that observed for WT EPEC, while the Δ*escS* strain transformed with pEscS_D46K_-HA or pEscS_K54A_-HA failed to support NleD effector translocation into HeLa cells and had no impact on JNK degradation (Figure 8B).

### Defective EscS proteins do not show a dominant-negative effect on T3SS activity

**Figure 9.**
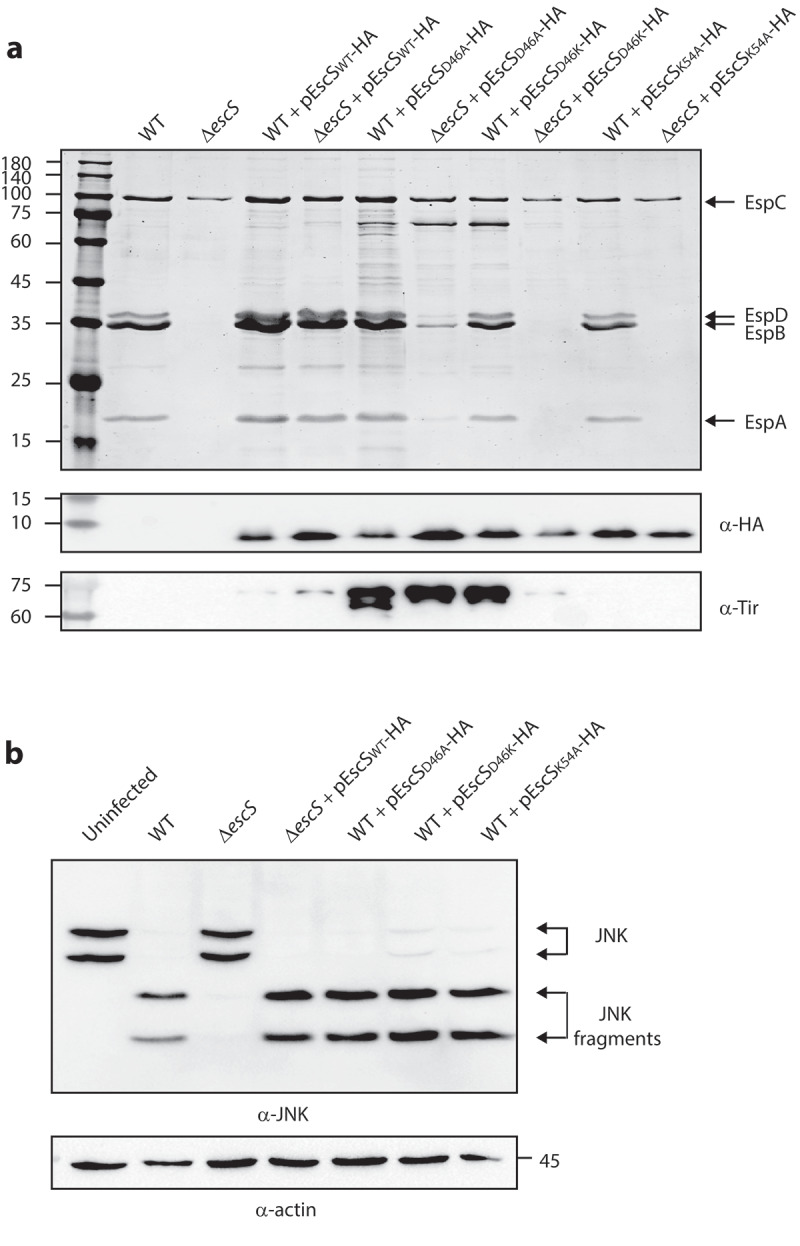
**EscS mutations do not have a dominant-negative effect on WT EPEC. (A)** Protein secretion profiles of WT EPEC transformed with EscS variants (D46A, D46K and K54A). The secreted fractions were obtained using a protocol similar to that described in the legend to Figure 2. The expression of EscS-HA variants was determined by analyzing the bacterial pellets on SDS-PAGE and western blot analysis with an anti-HA antibody (lower panel). The secretion of the Tir effector was assessed by analyzing the bacterial supernatant by western blotting with an anti-Tir antibody. **(B)** Proteins extracted from HeLa cells infected with WT, Δ*escS*, Δ*escS* expressing EscS_wt_-HA or WT EPEC expressing EscS_D46A_-HA, EscS_D46K_-HA or EscS_K54A_-HA were subjected to western blot analysis using anti-JNK and anti-actin (loading control) antibodies. JNK isoforms and their degradation fragments are indicated on the right of the gel

To examine the very plausible notion that expression of non-functional EscS variants would interfere with T3SS activity, we transformed pEscS-HA that carried a D46A, D46K or K54A mutation into WT EPEC. The secretion profile of WT EPEC transformed with pEscS_K54A_-HA was similar to that of WT EPEC, thus suggesting that the EscS_K54A_-HA copies did not inhibit T3SS activity (Figure 9A). We note that a similar lack of a dominant negative effect was previously observed for the E46A and K54A mutations in the FliQ flagellar protein.^29^ The secretion profile of WT EPEC transformed with pEscS_D46A_-HA or pEscS_D46K_-HA showed a similar secretion profile to that of WT EPEC but with the addition of Tir hypersecretion. Western blot analysis of the supernatants with anti-Tir antibody demonstrated enhanced Tir secretion for WT and Δ*escS* strains expressing the EscS_wt_-HA protein relative to EPEC WT (Figure 9A). However, much higher Tir secretion was observed in WT/Δ*escS* strains expressing EscS_D46A_-HA and the WT strain expressing EscS_D46K_-HA (Figure 9A). To examine whether these strains exhibited reduced ability to infect and facilitate translocation of effectors into the host cells, we infected HeLa cells with WT EPEC expressing EscS_D46A_-HA, EscS_D46K_-HA, or EscS_K54A_-HA. In keeping with the T3SS activity results, we observed that the expression of EscS mutant forms had no inhibitory effect on the ability of the WT strains to infect and facilitate translocation of effector proteins into the host cells (Figure 9B). These results suggest that either the mutant forms of EscS do not associate with the full T3SS complex or that complete replacement of all the EscS subunits is required for disruption of T3SS activity.

## Discussion

The core export apparatus complex (SctRST) of *Salmonella typhimurium* was reported to assemble into a funnel-like structure, consisting of six copies of SctT-like subunits, where the combined SctR and SctS structures mimic the structure of the SctT subunit.^8^ In its closed conformation, the complex contains three main constriction points, an M-gate and Q1- and Q2-belts, that are believed to function either as selective gates or as passive plugs that prevent ions and metabolites from leaking across membranes.^8,30^ The first structural confining element that a translocated substrate encounters upon its entry into the T3SS is the Q1-belt.^7–9^ The Q1-belt is formed from four conserved Gln-X-Gln-X-Gln motifs, each found in an SctS subunit (of which there are four). As shown by the findings presented in Figure 2, this study revealed a lack of interference with T3SS activity by single and double mutations of Gln to Ala, namely, Q39A, Q43A, Q45A, Q47A, and Q39A+Q47A (where a single mutation in an EscS will generate tetrameric mutations in the Q1-belt, since the complex contains four EscS subunits, and the double mutant will generate octameric mutations). We therefore concluded that the Q1-belt probably forms a mutation-resistant docking platform for T3SS substrates. This notion, in turn, suggests that regulation of substrate selection is determined prior to substrate arrival at the entrance of the export apparatus. Our results are in keeping with a previous study showing that mutations of the homologous residues in the FliQ protein of the flagellar system resulted in only a minor reduction of bacterial motility.^29^

Additional support for our premise that substrate selectivity is determined prior to substrate entrance into the export apparatus may be drawn from an examination of various mutations in the Q2-belt, which forms a loop that extends horizontally into the lumen of the channel and physically seals it.^8^ In this study, mutations in the loop had a fairly minor effect on T3SS activity and bacterial fitness (Figure 3). As it had been demonstrated that the loop enjoys conformational flexibility during substrate transport,^9^ we therefore aimed, in the current study, to limit this movement by replacing two residues in the loop with two large aromatic tryptophan residues. We observed that even this dramatic mutation (EscT_S115W+I116W_) had no effect on T3SS activity or bacterial fitness. These results are in keeping with recent structural data that indicated that the Q1-belt, the M-gate, and the Q2-belt form a number of control points that work in redundancy for sealing the channel during substrate transport to maintain membrane homeostasis.^8,9,30^ Therefore, mutations in a single channel constraint would not be sufficient to disrupt T3SS activity. Overall, our results thus suggest that the Q1- and Q2-belts are sequence-flexible components within the export apparatus and act as seals rather than as selective gates within the channel.

We also studied the tip of the EscRST complex, which is located adjacent to other T3SS substructures, such as the inner-membrane rings and the cytoplasmic complex (Figure 1). We previously demonstrated that replacing each of the two TMDs that form the tip of the EscS protein^8,9,30^ with a different hydrophobic sequence abolished T3SS activity.^18^ Here, we aimed to pinpoint the specific residues or motifs that are critical for T3SS activity by mutating aromatic, polar, and helix-breaker amino acids in the TMDs of EscS. Two mutations resulted in complete disruption of the secretion activity, namely, replacement with alanine of proline at position 23 and of lysine at position 54 (Figure 4). The high conservation of proline at position 23 and its characteristics as a helix breaker, which was consistently observed in the 3D structure of the *Salmonella* flagellum (PDB 6F2D), suggest that the kink in TMD1 is probably an important structural feature that serves to orient the tip of the export apparatus toward the T3SS cytoplasmic complex or to align the interaction interface of the EscRST subunits. Interestingly, replacement of the proline residue at position 50 had no effect on T3SS activity (Figure 4B). It is likely that Pro50 – being located at the edge of the hairpin region of EscS – does not bend the tip of the export apparatus and is therefore less crucial for T3SS activity.

The second mutation that induced complete disruption of the T3SS activity was K54A (Figure 4B). This finding is not novel, since Lys54 was previously reported to be critical for the activity of the flagellar- and virulence-T3SS of *Salmonella*, ^16,29^ forming an inter-subunit salt bridge with Asp46 in a neighboring subunit^8^ and facilitating the incorporation of the succeeding T3SS components into the assembling complex. ^16^ However, when we assessed the involvement of Lys54 in EscS self-association *in vitro*, we observed that the K54A mutation does not disrupt EscS self-interaction (Figure 5). This finding suggests that salt bridges between Lys54 and Asp46 are not essential for EscS self-interaction (which is apparently supported by many additional interactions between EscS subunits). This conclusion is in keeping with the FliPQR structure (PDB 6F2D), which showed various interactions between FliQ monomers. In addition, in analogy with the FliPQR 3D structure, we observed that while Lys54 of three EscS subunits interacts with Glu46 of a neighboring EscS subunit, Lys54 of the fourth EscS subunit interacts with the backbone of Thr214 of EscT. Unfortunately, we could not directly examine the involvement of Lys54 in the EscS-EscT interaction due to the very low expression level of EscT, even when it was expressed from an inducible expression vector (data not shown). To overcome this challenge, we co-expressed the three export apparatus proteins, EscRST, since co-expression has been reported to assist in stabilizing the proteins.^8,25^ Using this system, we demonstrated that EscS interacts with EscR and EscT (Figure 6). More importantly, we found that EscS with the K54A mutation abolished the EscS-EscT interaction but without disrupting the EscT-EscR interaction (Figure 6). This result indicates that while Lys54 is not crucial for EscS self-interaction, it is critical for the EscS-EscT interaction, and therefore it results in a T3SS loss of function.

To examine the role of the counter residue of the salt bridge, we mutated Asp46 to lysine and found that this mutation completely abolished T3SS activity (Figure 8). Based on previous reports demonstrating Asp46-Lys54 interactions between neighboring FliQ (EscS homolog) subunits, this loss of function of the D46K mutant can be explained simply in terms of the repulsion between two positively charged residues located on the interacting subunits. We also mutated Asp46 to alanine; in this case, the mutation caused dysregulation of substrate secretion (hypersecretion of the Tir effector and lower secretion of the translocators), thus suggesting that EscS is also involved in the mechanism responsible for the regulation of substrate secretion, which divides T3SS substrates into “early,” “intermediate,” and “late” secretion groups, as elaborated below (summarized in ref. ^1^). The early substrates are structural components that form the inner rod and the needle substructures of the T3SS. Following the completion of needle assembly, the T3SS switches to secretion of the intermediate substrates, which generate the filament extending from the bacterial membrane and the translocon complex that creates a pore within the host membrane. Only upon establishment of a continuous path into the host cell does the T3SS shift to secretion of effector proteins, which are considered late substrates. It is currently held that the regulation of substrate secretion, which is critical for the precise assembly of the T3SS and its timely response,^31–35^ involves the inner-membrane embedded export gate (SctV and SctU) ^7^ and the cytoplasmic complex, which acts as a substrate-sorting platform. As we had previously shown that no interactions were observed between EscS and EscV/EscU,^18^ we focused on the cytoplasmic complex. The cytoplasmic complex contains multiple SctQ subunits and an ATPase complex, which includes an ATPase (SctN), a negative regulator (SctL), and a positive regulator (SctO). The complex is not constantly anchored to the T3SS, but rather exchange between the cytoplasmic pool, where it recognizes T3SS chaperone-substrate pairs, and the membrane-anchored T3SS, where it loads the substrates for secretion.^25,36^ It has previously been suggested that two cytosolic proteins, SepL and SepD, are also involved in the regulation of the substrate secretion switch between translocators and effectors, and it has been found that deletion of either *sepL* or *sepD* abrogates the secretion of translocators and promotes the hypersecretion of effectors (we found that this phenotype is superior to the mutation in Asp46 of EscS; Supplementary Figure 4).^37^ Thus, to study whether D46A of EscS alters the regulation of substrate secretion through interaction with SepL and/or SepD or with components of the cytoplasmic complex, we examined the ability of EscS to interact with SepD, SepL, and EscQ *in vitro*. However, we could not detect stable interactions between EscS and these proteins (data not shown). To further examine and characterize the involvement of EscS in the regulation of substrate secretion, through interactions with the inner-membrane export apparatus proteins (EscV/EscU), the gatekeepers (SepL/D), or through interactions with the cytoplasmic-complex proteins, more sophisticated tools that allow better detection of transient interactions will be needed. Moreover, although the EscS_D46A_ mutant caused dysregulation of substrate secretion, it demonstrated similar JNK degradation to EPEC WT (Figure 8B). This result was unexpected, as previous studies reported that mutants with altered substrate secretion regulation demonstrated reduced ability to translocate effectors into host cells.^38–40^ A possible explanation for this phenotype is that either small amount of translocated NleD is sufficient for JNK degradation or that a shorter incubation period is required to ensnare the difference between the strains before JNK becomes a limiting factor.

In summary, in this study, we characterized the role of the EscRST complex within the T3SS complex. We found that the Q1- and Q2-belts formed by specific domains within the export apparatus channel constitute narrowed regions that are most probably responsible for preventing leakage of ions and metabolites during secretion through the T3SS complex. This observation suggests that regulation of substrate selection occurs prior to the entry of the substrates into the export apparatus. In addition, we showed that Lys54 of the EscS protein is crucial for EscS-EscT interaction and less significant for EscS self-oligomerization.

## Materials and methods

### Bacterial strains

Wild-type (WT) EPEC O127:H6 strain E2348/69 (streptomycin-resistant)^41^ and the EPEC null mutants, Δ*escN*, Δ*escS*, and Δ*escT*,^11,42^ were used to assess the T3SS and translocation activities. *E. coli* BL21 (λDE3) was used for protein expression, and *E. coli* DH10B was used for plasmid handling. The *E. coli* strains (Table 1) were grown at 37°C, unless otherwise indicated, in Luria-Bertani (LB) broth (Sigma) supplemented with the appropriate antibiotics. Bacterial growth was assessed by monitoring the absorbance at 600 nm of cultures grown in DMEM, without phenol red and with or without isopropyl β-d-1-thiogalactopyranoside (IPTG), for 6 h at 37°C. Antibiotics were used at the following concentrations: streptomycin (50 μg/mL), carbenicillin (100 μg/mL), and kanamycin (50 μg/mL).

**Table 1.** Strains and plasmids used in this study

Strain or plasmid	Description	Reference
Strain		
Wild-type EPEC	EPEC strain E2348/69 (streptomycin resistant)	^37^
EPEC ∆*escN*	Non-polar deletion of *escN*	^42^
EPEC ∆*escS*	Non-polar deletion of *escS*	^11^
EPEC ∆*escT*	Non-polar deletion of *escT*	^11^
*E. coli* DH10B	For plasmid handling	^43^
*E. coli* BL21 (λDE3)	For protein expression	Promega
**Plasmid**		
pEscS_wt_-HA (pSA10)	HA C-terminal tagged EscS in pSA10	^18^
pEscS_Q39A_-HA (pSA10)	HA C-terminal tagged EscS in pSA10 with a point mutation at position 39	This study
pEscS_Q39E_-HA (pSA10)	HA C-terminal tagged EscS in pSA10 with a point mutation at position 39	This study
pEscS_Q43A_-HA (pSA10)	HA C-terminal tagged EscS in pSA10 with a point mutation at position 43	This study
pEscS_Q43E_-HA (pSA10)	HA C-terminal tagged EscS in pSA10 with a point mutation at position 43	This study
pEscS_Q45A_-HA (pSA10)	HA C-terminal tagged EscS in pSA10 with a point mutation at position 45	This study
pEscS_Q45E_-HA (pSA10)	HA C-terminal tagged EscS in pSA10 with a point mutation at position 45	This study
pEscS_Q47A_-HA (pSA10)	HA C-terminal tagged EscS in pSA10 with a point mutation at position 47	This study
pEscS_Q47E_-HA (pSA10)	HA C-terminal tagged EscS in pSA10 with a point mutation at position 47	This study
pEscS_Q39A+Q47A_-HA (pSA10)	HA C-terminal tagged EscS in pSA10 with a double mutation at position 39 and 47	This study
pEscT_wt_-2HA (pSA10)	2 HA C-terminal tagged EscT in pSA10	^18^
pEscT_F112A_-2HA (pSA10)	2 HA C-terminal tagged EscT in pSA10 with a point mutation at position 112	This study
pEscT_N113A_-2HA (pSA10)	2 HA C-terminal tagged EscT in pSA10 with a point mutation at position 113	This study
pEscT_P114A_-2HA (pSA10)	2 HA C-terminal tagged EscT in pSA10 with a point mutation at position 114	This study
pEscT_D118A_-2HA (pSA10)	2 HA C-terminal tagged EscT in pSA10 with a point mutation at position 118	This study
pEscT_S115W+I116W_-2HA (pSA10)	2 HA C-terminal tagged EscT in pSA10 with a double point mutation at positions 115 and 116	This study
pEscS_F18A_-HA (pSA10)	HA C-terminal tagged EscS in pSA10 with a point mutation at position 18	This study
pEscS_P23A_-HA (pSA10)	HA C-terminal tagged EscS in pSA10 with a point mutation at position 23	This study
pEscS_G32V_-HA (pSA10)	HA C-terminal tagged EscS in pSA10 with a point mutation at position 32	This study
pEscS_G32I_-HA (pSA10)	HA C-terminal tagged EscS in pSA10 with a point mutation at position 32	This study
pEscS_G32F_-HA (pSA10)	HA C-terminal tagged EscS in pSA10 with a point mutation at position 32	This study
pEscS_S36A_-HA (pSA10)	HA C-terminal tagged EscS in pSA10 with a point mutation at position 36	This study
pEscS_D46A_-HA (pSA10)	HA C-terminal tagged EscS in pSA10 with a point mutation at position 46	This study
pEscS_D46K_-HA (pSA10)	HA C-terminal tagged EscS in pSA10 with a point mutation at position 46	This study
pEscS_P50A_-HA (pSA10)	HA C-terminal tagged EscS in pSA10 with a point mutation at position 50	This study
pEscS_K54A_-HA (pSA10)	HA C-terminal tagged EscS in pSA10 with a point mutation at position 54	^18^
pEscS_K54I_-HA (pSA10)	HA C-terminal tagged EscS in pSA10 with a point mutation at position 54	This study
pEscS_K54D_-HA (pSA10)	HA C-terminal tagged EscS in pSA10 with a point mutation at position 54	This study
pEscS_F59A_-HA (pSA10)	HA C-terminal tagged EscS in pSA10 with a point mutation at position 59	This study
pEscS_Y66A_-HA (pSA10)	HA C-terminal tagged EscS in pSA10 with a point mutation at position 66	This study
pEscS_wt_-V5 [pET28a(+)]	V5 C-terminal tagged EscS in pET28a(+)	^18^
pEscS_K54A_-V5 [pET28a(+)]	V5 C-terminal tagged EscS in pSA10 with a point mutation at position 54	This study
pEscR_wt_-3HA (pSA10)	3 HA C-terminal tagged EscR in pSA10	^11^
pEscR-3HA/EscS-V5/EscT-His (pSA10)	3 HA C-terminal tagged EscR, V5 C-terminal tagged EscS, His C-terminal tagged EscT in pSA10	This study
pEscR-3HA/EscS-V5/EscT (pSA10)	3 HA C-terminal tagged EscR, V5 in C-terminal tagged EscS, no tag EscT in pSA10	This study
pEscR-3HA/EscS_P23A_-V5/EscT-His (pSA10)	3 HA C-terminal tagged EscR, V5 C-terminal tagged EscS with a point mutation at position 23, His C-terminal tagged EscT in pSA10	This study
pEscR-3HA/EscS_K54A_-V5/EscT-His (pSA10)	3 HA C-terminal tagged EscR, V5 C-terminal tagged EscS with a point mutation at position 54, His C-terminal tagged EscT in pSA10	This study
pEscR-3HA/EscS_D46A_-V5/EscT-His (pSA10)	3 HA C-terminal tagged EscR,V5 C-terminal tagged EscS with a point mutation at position 46, His C-terminal tagged EscT in pSA10	This study
pEscR-3HA/EscS_D46K_-V5/EscT-His (pSA10)	3 HA C-terminal tagged EscR,V5 C-terminal tagged EscS with a point mutation at position 46, His C-terminal tagged EscT in pSA10	This study

### Construction of a plasmid co-expressing labeled EscR, EscS, and EscT

The *escS* gene, tagged with V5, was amplified from pEscS-V5 (pET28)^18^ by using the primer pair EscS_F_com/EscS_R_com (Table 2). The 3HA-labeled *escR* gene was amplified from pEscR-3HA (pSA10) ^11^ by using the primer pair EscR_F/EscR_R (Table 2). The *escT* gene was amplified from the EPEC genome and tagged with a His tag by using the primer pairs EscT_F_com/EcsT_His_R1 and then EscT_F_com/EcsT_His_R2_pSA10 (Table 2). The PCR fragments were fused using PCR to form an EscR-3 HA-EscS-V5-EscT-His fragment. The pSA10 plasmid was amplified using the primer pair pSA10_F/pSA10_R. The open plasmid and the fused PCR product were digested with *Dpn*I, purified, and assembled by the Gibson assembly method.^44–46^ The resulting construct, pEscR-3HA/EscS-V5/EscT-His in pSA10 (designated pEscRST in this article), expressed a full-length EscR protein fused to a C-terminus triple HA tag, a full-length EscS protein fused to a C-terminal V5 tag, and a full-length EscT protein fused to a C-terminal His tag.

**Table 2.** Sequences of primers designed and used in this study

Plasmid	Primer name	Primer sequence
pEscS_Q39A_-HA (pSA10)	EscS_Q39A_F	TAGTCTGGTCGCGGCTATAACGCAGTTACAG
	EscS_Q39A_R	GCGTTATAGCCGCGACCAGACTAATAATAATACCGATAACAG
pEscS_Q39E_-HA (pSA10)	EscS_Q39E_F	AGTCTGGTCGAGGCTATAACGCAGTTACAG
	EscS_Q39E_R	GTTATAGCCTCGACCAGACTAATAATAATACCGATAACAG
pEscS_Q43A_-HA (pSA10)	EscS_Q43A_F	GGCTATAACGGCGTTACAGGATCAAACATTGCC
	EscS_Q43A_R	GATCCTGTAAGCGCGTTATAGCCTGGACCAGAC
pEscS_Q43E_-HA (pSA10)	EscS_Q43E_F	GGCTATAACGGAGTTACAGGATCAAACATTGCC
	EscS_Q43E_R	GATCCTGTAAGTGCGTTATAGCCTGGACCAGAC
pEscS_Q45A_-HA (pSA10)	EscS_Q45A_F	ATAACGCAGTTAGCGGATCAAACATTGCCTTTTTTGC
	EscS_Q45A_R	CAATGTTTGATCCGCTAACTGCGTTATAGCCTGGAC
pEscS_Q45E_-HA (pSA10)	EscS_Q45E_F	ATAACGCAGTTAGAGGATCAAACATTGCC
	EscS_Q45E_R	ATGTTTGATCCTCTAACTGCGTTATAGCCTG
pEscS_Q47A_-HA (pSA10)	EscS_Q47A_F	CAGTTACAGGATGCAACATTGCCTTTTTTGCTAAAAATAATAGC
	EscS_Q47A_R	AAAAGGCAATGTTGCATCCTGTAACTGCGTTATAGC
pEscS_Q47E_-HA (pSA10)	EscS_Q47E_F	CAGTTACAGGATGAAACATTGCCTTTTTTGC
	EscS_Q47E_R	AAGGCAATGTTTCATCCTGTAACTGCGTTATAGC
pEscT_wt_-2HA (pSA10)	EscT_F	CAATTTCACACAGGAAACAGATGAATGAGATAATGACGG
	EscT_2 HA_R1	GGTAAGCGTAATCTGGAACATCGTATGGGTACTCATTAATCATG CTCGG
	EscT_2 HA_R2	GATCCCCGGGAATTTCAAGCGTAATCTGGAACATCGTATGGGTA AGCGTAATCTGG
pEscT_F112A_-2HA (pSA10)	EscT_F112A_F	ATATCTTCAATTGCTAACCCGTCCATAAGTGATTC
	EscT_F112A_R	GGACGGGTTAGCAATTGAAGATATTGTTGAACCTCTTAG
pEscT_N113A_-2HA (pSA10)	EscT_N113A_F	ATATCTTCAATTTTTGCCCCGTCCATAAGTGATTC
	EscT_N113A_R	GAATCACTTATGGACGGGGCAAAAATTGAAGATAT
pEscT_P114A_-2HA (pSA10)	EscT_P114V_F	CAATTTTTAACGCGTCCATAAGTGATTCATCTTC
	EscT_P114V_R	CTTATGGACGCGTTAAAAATTGAAGATATTGTTGAACC
pEscT_D118A_-2HA (pSA10)	EscT_D118A_F	CCCGTCCATAAGTGCTTCATCTTCTATCACTGGCG
	EscT_D118A_R	AGAAGATGAAGCACTTATGGACGGGTTAAAAATTG
pEscT_S115W+I116W_-2HA (pSA10)	EscT_S115W+I116W_F	TTTTAACCCGTGGTGGAGTGATTCATCTTCTATCACTG
	EscT_S115W+I116W_R	GATGAATCACTCCACCACGGGTTAAAAATTGAAGATATTG
pEscS_F18A_-HA (pSA10)	EscS_F18A_F	TGGATAATAGCTATCCTCTCATTGCCTACAG
	EscS_F18A_R	CAATGAGAGGATAGCTATTATCCAGAACGTTTGC
pEscS_P23A_-HA (pSA10)	EscS_P23A_F	TTTATCCTCTCATTGGCTACAGTCATAGCG
	EscS_P23A_R	CGCTATGACTGTAGCCAATGAGAGGATAAA
pEscS_G32V_-HA (pSA10)	EscS_G32V_F	CTCTGTTATCGTTATTATTATTAGTCTGGTCCAGGC
	EscS_G32V_R	CTAATAATAATAACGATAACAGAGGCCGCTATGAC
pEscS_G32I_-HA (pSA10)	EscS_G32I_F	CTCTGTTATCATTATTATTATTAGTCTGGTCCAGGC
	EscS_G32I_R	CTAATAATAATAATGATAACAGAGGCCGCTATGAC
pEscS_G32F_-HA (pSA10)	EscS_G32F_F	CTCTGTTATCTTTATTATTATTAGTCTGGTCCAGGC
	EscS_G32F_R	CTAATAATAATAAAGATAACAGAGGCCGCTATGAC
pEscS_S36A_-HA (pSA10)	EscS_S36A_F	GTTATCGGTATTATTATTGCTCTGGTCCAGGCT
	EscS_S36A_R	AGCCTGGACCAGAGCAATAATAATACCGATAAC
pEscS_D46A_-HA (pSA10)	EscS_D46A_F	GCTATAACGCAGTTACAGGCTCAAACATTGCCTTTTTTGC
	EscS_D46A_R	GCAAAAAAGGCAATGTTTGAGCCTGTAACTGCGTTATAGC
pEscS_D46K_-HA (pSA10)	EscS_D46K_F	GCTATAACGCAGTTACAGAAACAAACATTGCCTTTTTTGC
	EscS_D46K_R	GCAAAAAAGGCAATGTTTGTTTCTGTAACTGCGTTATAGC
pEscS_P50A_-HA (pSA10)	EscS_P50A_F	CATTGGCTTTTTTGCTAAAAATAATAGCAGTG
	EscS_P50A_R	ATTTTTAGCAAAAAAGCCAATGTTTGATCCTG
pEscS_K54A_-HA (pSA10)	EscS_K54A_F	TTGCCTTTTTTGCTAGCAATAATAGCAGTGTTTGCT
	EscS_K54A_R	AGCAAACACTGCTATTATTGCTAGCAAAAAAGGCAA
pEscS_K54I_-HA (pSA10)	EscS_K54I_F	TTGCCTTTTTTGCTAATAATAATAGCAGTGTTTGCT
	EscS_K54I_R	AGCAAACACTGCTATTATTATTAGCAAAAAAGGCAA
pEscS_K54D_-HA (pSA10)	EscS_K54D_F	TTGCCTTTTTTGCTAGACATAATAGCAGTGTTTGCT
	EscS_K54D_R	AGCAAACACTGCTATTATGTCTAGCAAAAAAGGCAA
pEscS_F59A_-HA (pSA10)	EscS_F59A_F	ATAATAGCAGTGGCTGCTACGCTTGCCCTG
	EscS_F59A_R	CAAGCGTAGCAGCCACTGCTATTATTTTTAGCAAAAAAGGC
pEscS_Y66A_-HA (pSA10)	EscS_Y66A_F	CCCTGACTGCTCACTGGATGGGAACAACAATC
	EscS_Y66A_R	CCCATCCAGTGAGCAGTCAGGGCAAGCGTAGC
pEscS_K54A_-V5 (pET28)	EscS_K54A_F	TTGCCTTTTTTGCTAGCAATAATAGCAGTGTTTGCT
	EscS_K54A_R	AGCAAACACTGCTATTATTGCTAGCAAAAAAGGCAA
pEscR-3HA/EscS-V5/EscT-His (pSA10)	pSA10_F	AATTCCCGGGGATCCGTCG
	pSA10_R	CTGTTTCCTGTGTGAAATTGTTATCCG
	EscS_F_com	CCGTCGAGAAGGAGATATACATATGGATACTGGATATTTTGTTC
	EscS_R_com	CCCTCCTTTACGTAGAATCGAGACCGAG
	EscT_F_com	ATTCTACGTAAAGGAGGGCACGTTAATGAATGAGATAATGACGG
	EcsT_His_R1	GTGGTGGTGGTGGTGGTGCTCATTAATCATGCTCGG
	EcsT_His_R2_pSA10	GGATCCCCGGGAATTTCAGTGGTGGTGGTGGTGGTG
	EscR_F	TTTCACACAGGAAACAGATGTCTCAATTAATGACCATTGGCTCAC
	EscR_R	ATGTATATCTCCTTCTCGACGGATCCCCGGG
pEscR-3HA/EscS-V5/EscT (pSA10)	EscT-stop_F	CCGAGCATGATTAATGAGTAACACCACCACCAC
	EscT-stop_R	GTGGTGGTGGTGTTACTCATTAATCATGCTCGG
pEscR-3HA/EscS_P23A_-V5/EscT (pSA10)	EscS_P23A_F	TTTATCCTCTCATTGGCTACAGTCATAGCG
	EscS_P23A_R	CGCTATGACTGTAGCCAATGAGAGGATAAA
pEscR-3HA/EscS_K54A_-V5/EscT (pSA10)	EscS_K54A_F	TTGCCTTTTTTGCTAGCAATAATAGCAGTGTTTGCT
	EscS_K54A_R	AGCAAACACTGCTATTATTGCTAGCAAAAAAGGCAA
pEscR-3HA/EscS_D46A_-V5/EscT (pSA10)	EscS_D46A_F	GCTATAACGCAGTTACAGGCTCAAACATTGCCTTTTTTGC
	EscS_D46A_R	GCAAAAAAGGCAATGTTTGAGCCTGTAACTGCGTTATAGC
pEscR-3HA/EscS_D46K_-V5/EscT (pSA10)	EscS_D46K_F	GCTATAACGCAGTTACAGAAACAAACATTGCCTTTTTTGC
	EscS_D46K_R	GCAAAAAAGGCAATGTTTGTTTCTGTAACTGCGTTATAGC

### Site-directed mutagenesis

Site-directed mutagenesis of Q39A, Q39E, Q43A, Q43E, Q45A, Q45E, Q47A, Q47E, F18A, P23A, G32V, G32I, G32F, S36A, D46A, D46K, P50A, K54I, K54D, F59A, and Y66A in the EscS-HA (pSA10) construct was performed using the primer pairs EscS_Q39A_F/EscS_Q39A_R, EscS_Q39E_F/EscS_Q39E_R, EscS_Q43A_F/EscS_Q43A_R, EscS_Q45A_F/EscS_Q45A_R, EscS_Q45E_F/EscS_Q45E_R, EscS_Q47A_F/EscS_Q47A_R EscS_Q47E_F/EscS_Q47E_R, EscS_F18A_F/EscS_F18A_R, EscS_P23A_F/EscS_P23A_R, EscS_G32V_F/EscS_G32V_R, EscS_G32I_F/EscS_G32I_R, EscS_G32F_F/EscS_G32F_R, EscS_S36A_F/EscS_S36A_R, EscS_D46A_F/EscS_D46A_R, EscS_D46K_F/EscS_D46K_R, EscS_P50A_F/EscS_P50A_R, EscS_K54I_F/EscS_K54I_R, EscS_K54D_F/EscS_K54D_R, EscS_F59A_F/EscS_F59A_R EscS_Y66A_F/EscS_Y66A_R, respectively (Table 2). The double mutant EscSQ39A+Q47A was constructed on EscSQ39A-HA (pSA10) using the primer pair EscS_Q47A_F/EscS_Q47A_R. Site-directed mutagenesis of F112A, N113A, P114A, D118A and S115W+I116W in the EscT-2 HA (pSA10) construct was performed using the primer pairs EscT_F112A_F/EscT_F112A_R, EscT_F113N_F/EscT_N113A_R, EscT_P114A_F/EscT_P114A_R, EscT_D118A_F/EscT_D118A_R and EscT_S115W+I116W_F/EscT_S115W+I116W_R, respectively (Table 2). Site-directed mutagenesis of K54A in EscS-V5 encoded on the pET28a(+) vector was performed using the primer pair EscS_K54A_F/EscS_K54A_R (Table 2). Site-directed mutagenesis of P23A, D46A, D46K, and K54A in the EscR-3 HA-EscS-V5-EscT-His (pSA10) construct was performed using the primer pairs EscS_P23A_F/EscS_P23A_R, EscS_D46A_F/EscS_D46A_R, EscS_D46K_F/EscS_D46K_R and EscS_K54A_F/EscS_K54A_R, respectively (Table 2). The vector pEscR-3HA/EscS-V5/EscT (pSA10), in which the EscT protein is not tagged, was generated by site-directed mutagenesis in the pEscR-3HA/EscS-V5/EscT-His (pSA10) construct by using the primer pair EscT-stop_F/EscT-stop_R (Table 2) to introduce a stop codon before the His tag. The PCR products were digested with *Dpn*I, purified, and transformed in competent bacteria. All point mutations were verified by DNA sequencing.

### In vitro type III secretion (T3S) assay

T3S assays were performed as previously described.^18,39^ Briefly, EPEC strains were grown overnight in LB, supplemented with the appropriate antibiotics, in a shaker at 37°C. The cultures were then diluted 1:40 into pre-heated Dulbecco’s modified Eagle’s medium (DMEM, Biological Industries), supplemented with the appropriate antibiotics, and grown statically for 6 h in a tissue-culture incubator (with 5% CO_2_) to an optical density of 0.7 at 600 nm (OD_600_). To induce protein expression, 0.25 mM IPTG was added to the bacterial cultures. The cultures were then centrifuged at 20,000 × g for 5 min; the bacterial pellet was dissolved in SDS-PAGE sample buffer; and the supernatant, containing the secreted proteins, was collected and filtered through a 0.22-μm filter (Millipore). The supernatants were normalized according to the bacterial OD_600_ and precipitated with 10% (v/v) trichloroacetic acid (TCA) overnight at 4°C to concentrate the proteins secreted into the culture medium. The samples were then centrifuged at 18,000 × g for 30 min at 4°C; the precipitates of the secreted proteins were dissolved in SDS-PAGE sample buffer; and the residual TCA was neutralized with saturated Tris. Proteins were analyzed on SDS-PAGE gels with Coomassie Blue straining.

### Immunoblotting

Samples were subjected to SDS-PAGE and transferred to nitrocellulose (pore size: 0.45 μm; Amersham Protran) or PVDF (pore size: 0.45 μm; Amersham Hybond) membranes. The blots were then blocked for 1 h with 5% (w/v) skim milk-PBST (0.1% Tween in phosphate-buffered saline), incubated with the primary antibody (diluted in 5% skim milk-PBST, for 1 h, at room temperature, unless otherwise indicated), washed, and then incubated with the secondary antibody (diluted in 5% skim milk-PBST, for 1 h, at room temperature). Chemiluminescence was detected with EZ-ECL reagents (Biological Industries). The following primary antibodies were used: mouse anti-HA (Abcam Inc.), diluted 1:1,000; mouse anti-HA.11 (Covance), diluted 1:1,000; mouse anti-V5 (Invitrogen), diluted 1:1,000; mouse anti His (Pierce), diluted 1:2000; mouse anti-JNK (BD Pharmingen), diluted 1:1,000 in Tris-buffered saline (TBS); and mouse anti-actin (MPBio), diluted 1:10,000. Antibodies directed against T3SS components included mouse anti-EspA, mouse anti-EspB and mouse anti-Tir, all a generous gift from Prof. B. Brett Finlay (University of British Columbia, Canada). Horseradish peroxidase-conjugated (HRP)-goat anti-mouse (Abcam Inc.), diluted 1:10,000, was used as the secondary antibody. Representative western blots of at least three independent experiments are presented in the results section.

### Co-immunoprecipitation

*E. coli* BL21 (λDE3) transformed with pEscS_wt_-HA, pEscS_wt_-V5, pEscS_K54A_-HA or pEscS_K54A_-V5 was grown to mid-exponential phase in LB and induced with 0.25 mM IPTG (6 h, 37°C). Cells were then harvested by centrifugation (4,000 × *g*, 30 min, 4°C) and washed twice with PBS. The washed pellets were resuspended in lysis buffer (20 mM Tris-HCl pH 7.5, 150 mM NaCl, 3 mM MgCl_2_, 1 mM CaCl_2_, and 2 mM 2-mercaptoethanol with a protease inhibitor cocktail), sonicated (Fisher Scientific, 3 × 15 s), and then incubated with 1% n-dodecyl-β-D-maltoside (DDM), at 4°C with rotation for 60 min. Intact cells were removed by centrifugation (18,000 × *g*, 15 min, 4°C). Whole-cell lysates (WCLs) were collected and aliquoted into tubes containing only EscSwt-HA, EscSwt-V5, EscS_K54A_-HA, or EscS_K54A_-V5 lysates or HA/V5 combinations. Samples were topped up to the same volumes by adding lysis buffer. Mouse anti-HA.11 antibody (1.5 μg) was then added to all samples, which were incubated for 30 min at 4°C on a rotary wheel. Thereafter, washed protein G slurry beads were added to each sample, followed by incubation on a rotatory wheel overnight at 4°C. Finally, the beads were centrifuged, washed five times with 1 mL of lysis buffer, and eluted by adding SDS-PAGE sample buffer and boiling the beads for 10 min. Equal amounts of WCLs and eluted fractions were subjected to SDS-PAGE and then western blot analysis with anti-HA and anti-V5 antibodies.

### Pull-down assay

The pull-down assay under native conditions was performed according to the QIAGEN handbook (https://www.qiagen.com/au/resources/resourcedetail?id=79ca2f7d-42fe-4d62-8676-4cfa948c9435&lang=en). *E. coli* BL21 (λDE3) cells carrying the pEscR-3HA-EscS-V5-EscT-His, pEscR-3HA-EscS-V5-EscT or pEscR-3HA-EscS_K54A_-V5-EscT-His plasmid were grown to mid-exponential phase in LB and induced with 0.25 mM IPTG (18 h, 16°C). Cells were harvested by centrifugation (4,000 × *g*, 30 min, 4°C) and washed twice with PBS. The washed pellets were resuspended in lysis buffer, sonicated (Fisher Scientific, 3 × 15 s) and then incubated with 0.1% Nonidet P-40 (NP-40), on ice, for 15 min. Intact cells were removed by centrifugation (18,000 × *g*, 15 min, 4°C). WCLs were collected, aliquoted into tubes containing washed Ni-NTA beads (Adar Biotech), and incubated on a rotatory wheel overnight at 4°C. The next day, the beads were centrifuged, washed five times with 1 mL of lysis buffer, and eluted by adding SDS-PAGE sample buffer and boiling the beads for 10 min. Equal amounts of WCLs and eluted fractions were subjected to SDS-PAGE and then western blot analysis with anti-HA, anti-V5, and anti-His antibodies. For pull down, under native conditions and in the context of the complete T3SS, cultures of EPEC Δ*escS* were grown in the absence or the presence of an pEscRST plasmid containing either the WT sequence of EscS or EscS with the point mutation P23A or K54A. The strains were grown for 7 h under T3SS-inducing conditions and induced with 0.25 mM IPTG. Cells were harvested using a protocol similar to that described for the co-immunoprecipitation assay, with the exception that the modified lysis buffer did not contain 2-mercaptoethanol. WCLs were incubated overnight with NTA-Ni beads, centrifuged, washed five times with 1 mL of modified lysis buffer (containing 30 mM imidazole), eluted with 500 mM imidazole in lysis buffer, and incubated on a rotatory wheel for 15 min. The samples were then subjected to SDS-PAGE and western blot analysis.

### Translocation assay

Translocation assays were performed as previously described.^28^ Briefly, HeLa cells (8 × 10^5^ cells per well) were infected for 3 h with EPEC strains that had been pre-induced for 3 h for T3SS activity (pre-heated DMEM, statically, in a CO_2_ tissue culture incubator). Cells were then washed with cold PBS, collected, and lysed with RIPA buffer. Thereafter, samples were centrifuged at 18,000 × g for 5 min to remove non-lysed cells, and supernatants were collected, mixed with SDS-PAGE sample-buffer, and subjected to western blot analysis with anti-JNK and anti-actin antibodies (loading control). Uninfected samples and samples infected with the Δ*escN* mutant strain were used as negative controls.

## Supplementary Material

Supplemental Material

## Data Availability

The authors confirm that the data supporting the findings of this study are available within the article.
