# Supplementary material for: Interactions and substrate selectivity within the SctRST complex of the type III secretion system of enteropathogenic Escherichia coli
Source: Gut Microbes. 2021 Dec 29;14(1):2013763. doi: 10.1080/19490976.2021.2013763 (PMC8726614; doi:10.1080/19490976.2021.2013763)
Supplement: Supplemental Material [file KGMI_A_2013763_SM6101.zip › supplementary/Supplementary_material_EscRST_final.docx]

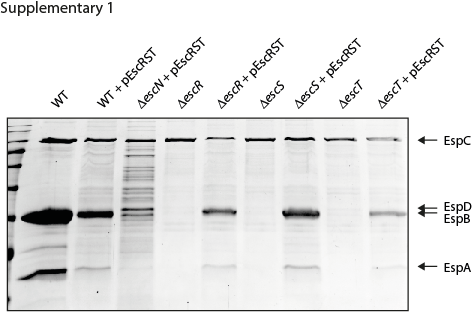


Supplementary Figure 1: **Co-expression of labeled EscR, EscS, and EscT proteins encode functional proteins**. Protein secretion profiles of WT EPEC, Δ*escN,* Δ*escR,* Δ*escS,* Δ*escT* with and without the pEscRST vector. The strains were grown under T3SS-inducing conditions and protein expression was induced with IPTG. The secreted fractions were normalized, filtered, protein content was concentrated from the supernatants of bacterial cultures and analyzed by 12% SDS-PAGE and Coomassie staining. The T3SS-secreted translocators EspA, EspB, and EspD are marked on the right of the gel. Also indicated is the location of EspC, which is not secreted via the T3SS. Expression of EscRST vector with the Δ*escR,* Δ*escS*, and Δ*escT* restored T3SS activity while expressing of EscRST within the Δ*escN* strain, had no T3SS activity.


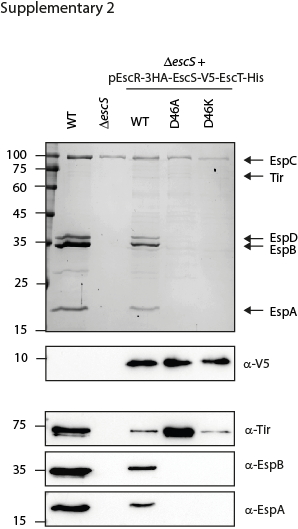


Supplementary Figure 2: **Point mutation at Asp46 within EscS loop results in impaired type III substrate secretion**. Protein secretion profiles of WT EPEC, Δ*escS*, and Δ*escS* expressing either EscRST, EscRS_D46A_T, or EscRS_D46K_T. The secreted fractions were obtained using a similar protocol to that described in the legend to a Supplementary Fig. 1. The expression of EscS-V5 variants was identified by analyzing the bacterial pellets on SDS-PAGE and western blot analysis with an anti-V5 antibody. The Δ*escS* strain expressing the WT EscS sequence (EscRST) restored T3 secretion, while expression of EscS with D46A point mutation resulted in dysregulated T3S and expression of EscS with D46K abolished T3S.


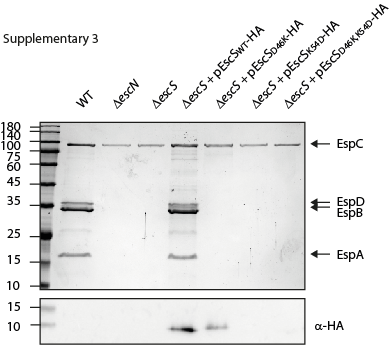


Supplementary Figure 3: **Mutations of the charged residues Asp46 and Lys54** **of EscS**. Protein secretion profiles of WT EPEC, Δ*escN,* Δ*escS*, and Δ*escS* expressing EscS_WT_-HA, EscS_D46K_-HA, EscS_K54D_-HA, or the double mutant EscS_D46K, K54D_-HA. The secreted fractions were obtained using a similar protocol to that described in the legend to Supplementary Fig. 1. The expression of EscS-HA variants was identified by analyzing the bacterial pellets on SDS-PAGE and by western blot analysis with an anti-HA antibody. The Δ*escS* strain expressing the WT EscS sequence (EscS_WT_-HA) restored T3 secretion, while expression of EscS with D46K abolished T3S. The Δ*escS* strain expressing either EscS_K54D_-HA or EscS_D46K_,_K54D_-HA showed no EscS expression, and therefore conclusions regarding the lack of T3SS activity cannot be drawn.


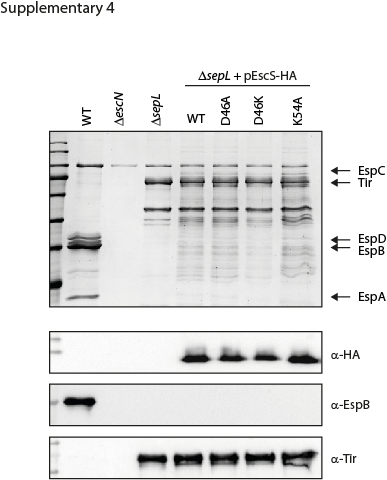


Supplementary Figure 4: **EscS mutations do not have an additive effect on the Δ*sepL* T3SS pattern**. Protein secretion profiles of WT EPEC, Δ*escN,* Δ*sepL*, and Δ*sepL* expressing EscS_WT_-HA, EscS_D46A_-HA, EscS_D46K_-HA, or EscS_K54A_-HA. The secreted fractions were obtained using a similar protocol to that described in the legend to Supplementary Fig. 1. The expression of EscS-HA variants was identified by analyzing the bacterial pellets on SDS-PAGE and western blot analysis with an anti-HA antibody, and the level of Tir and EspB secretion was determined by analyzing the normalized bacterial supernatants on SDS-PAGE and western blot analysis with anti-EspB and anti-Tir antibodies. All Δ*sepL* variants showed similar T3SS secretion patterns.
